# The intellectual base and global trends in contrast-induced acute kidney injury: a bibliometric analysis

**DOI:** 10.1080/0886022X.2023.2188967

**Published:** 2023-03-17

**Authors:** Heng Wang, Tingting Gao, Ruijing Zhang, Jie Hu, Yuwen Wang, Jianing Wei, Yun Zhou, Honglin Dong

**Affiliations:** a Department of Vascular Surgery, The Second Hospital of Shanxi Medical University, Taiyuan, China; b Department of Nephrology, The Second Hospital of Shanxi Medical University, Taiyuan, China; c Key Laboratory of Cardiovascular Disease Diagnosis, Treatment and Clinical Pharmacology of Shanxi Province, The Second Hospital of Shanxi Medical University Cardiovascular Medicine, Taiyuan, China; d Shanxi Province Integrated Traditional and Western Medicine Hospital, Taiyuan, China

**Keywords:** Contrast-induced acute kidney injury, bibliometric analysis, CiteSpace, VOSviewer, research frontier

## Abstract

Contrast-induced acute kidney injury (CI-AKI) has become the third leading cause of hospital-acquired kidney injury. A comprehensive analysis of the current state of research in the field of CI-AKI will help to reveal trends and hot topics in the field. To date, there are no published bibliometric analyses related to CI-AKI studies. Here, we analyze the relevant literature since the emergence of the concept and provide valuable insights. The literature was collected from the Web of Science Core Collection. The data were analyzed visually using CiteSpace and VOSviewer software. We collected a total of 4775 papers, with the United States and Guangdong Acad Med Sci as the major publishing powers in terms of country/region and institution. J AM COLL CARDIOL was the journal with the most published and cocited articles. Cluster analysis showed that clinical trials are the current research hotspot. The areas of risk assessment, prevention strategies, risk factors, and vascular lesions have been popular in recent years. Research on the mechanism of injury in CI-AKI will be the focus of future research, which will be crucial to reduce the clinical incidence of CI-AKI. In summary, this study provides a comprehensive analysis of the development process in the field of CI-AKI and discusses future research directions based on the analysis of objective data from many studies on CI-AKI.

## 1. Introduction

In recent years, with the continuous development of imaging CT technology and intracavitary cardiovascular and peripheral vascular interventional techniques, the application of contrast medium (CM) has become increasingly frequent, and contrast-induced acute kidney injury (CI-AKI) has become a research hotspot in the fields of nephrology, radiology, cardiovascular and peripheral vasculature [1–4]. The definition of CI-AKI given by the Kidney Disease: Improving Global Outcomes (KDIGO) organization is a rise in serum creatinine (SCr) of ≥0.5 mg/dL (≥44 μmol/L) or a 25% increase from baseline, assessed at 48 h after a radiological procedure [5,6]. CI-AKI occurs in up to 30% of patients receiving iodine contrast and is often considered to be the third leading cause of hospital-acquired kidney injury [7,8]. The causal relationship between contrast and AKI has not been strictly established, but rather the AKI is called contrast-associated acute kidney injury (CA-AKI). In particular, the prevalence in high-risk patients with advanced age, diabetes, chronic kidney disease (CKD), and hypertensive disease is 40%, of whom ∼11% develop end-stage renal disease [5,9–11]. In addition, acute kidney injury often leads to multiorgan damage, but the exact mechanism of injury has not been clarified [12]. The pathophysiological mechanisms of CI-AKI are not fully understood. Three main mechanisms of CI-AKI are known, including direct tubular toxic effects, intrarenal vasoconstriction, and excessive generation of reactive oxygen species (ROS) [13]. The key pathophysiological mechanisms underlying the development of CI-AKI are direct cytotoxic effects and hemodynamic alterations leading to renal hypoperfusion [14,15]. Oxidative stress and hypoxia-mediated direct cytotoxic effects mainly damage renal tubular epithelial and endothelial cells [16,17]. The hemodynamic changes are the result of vasoconstriction and indirect damage to the peritubular vascular endothelium *via* reactive oxygen species [18,19]. CI-AKI has become an important medical complication that endangers human health, but restricting the use of CM in high-risk groups has become a barrier to clinical diagnosis and treatment.

Currently, most of the research on CI-AKI is focused on clinical trials and molecular mechanisms. Review articles account for a significant proportion of articles but are more focused on a limited number of aspects, such as meta-analyses exploring risk factors, practice guidelines, and traditional reviews [20–22]. These articles suffer from a lack of integration and limited reproducibility. Bibliometrics is an effective method for describing trends in research fields and is widely used in medical research [23,24]. CiteSpace and VOSviewer are software programs that facilitate visual analysis of new trends in scientific development, helping researchers quickly identify existing scientific progress and discover and track research hotspots [25,26]. The purpose of this study was to qualitatively and quantitatively analyze the significant, reliable, and comprehensive information in the CI-AKI field over the last 40 years since the emergence of reports on this topic. We hope to gain a deeper understanding of current hot spots and to generate intense discussion of potential research directions.

## 2. Materials and methods

### 2.1. Data source and search strategy

The literature used in this paper is drawn from the core collection database of the WoS website (WoSCC), as this is considered one of the most systematic, authoritative, and comprehensive databases and is widely used in bibliometric and visual analyses [27,28]. All data were independently searched and downloaded by the two authors on 25 August 2022, after which no raw data were downloaded from the database. The effect of daily updates to the database on the articles was ignored. The search strategy was as follows: (TS = (Contrast-induced acute kidney injury) OR TS = (Contrast-associated acute kidney injury) OR TS = (Contrast-induced nephropathy)) AND LA = (English). The selection criteria were as follows: (i) language: English; (ii) document type: all types; (iii) time span: up to 25 August 2022; (iv) document format: plain text; and (v) record content: full record with cited references. A total of 4775 documents were retrieved.

### 2.2. Data analysis and visualization

CiteSpace 6.1 R3 and VOSviewer 1.6.18 were used to build a visual bibliometric analysis based on collaborative networks, co-word analysis, and cocitation analysis. The information used includes authors’ names, institutions, nationalities, journals, keywords, references, etc., and these important scientific terms are visualized by constructing a co-occurrence network.

## 3. Results

### 3.1. Global publishing characteristics

#### 3.1.1. Article volume characteristics

**Figure 1.**
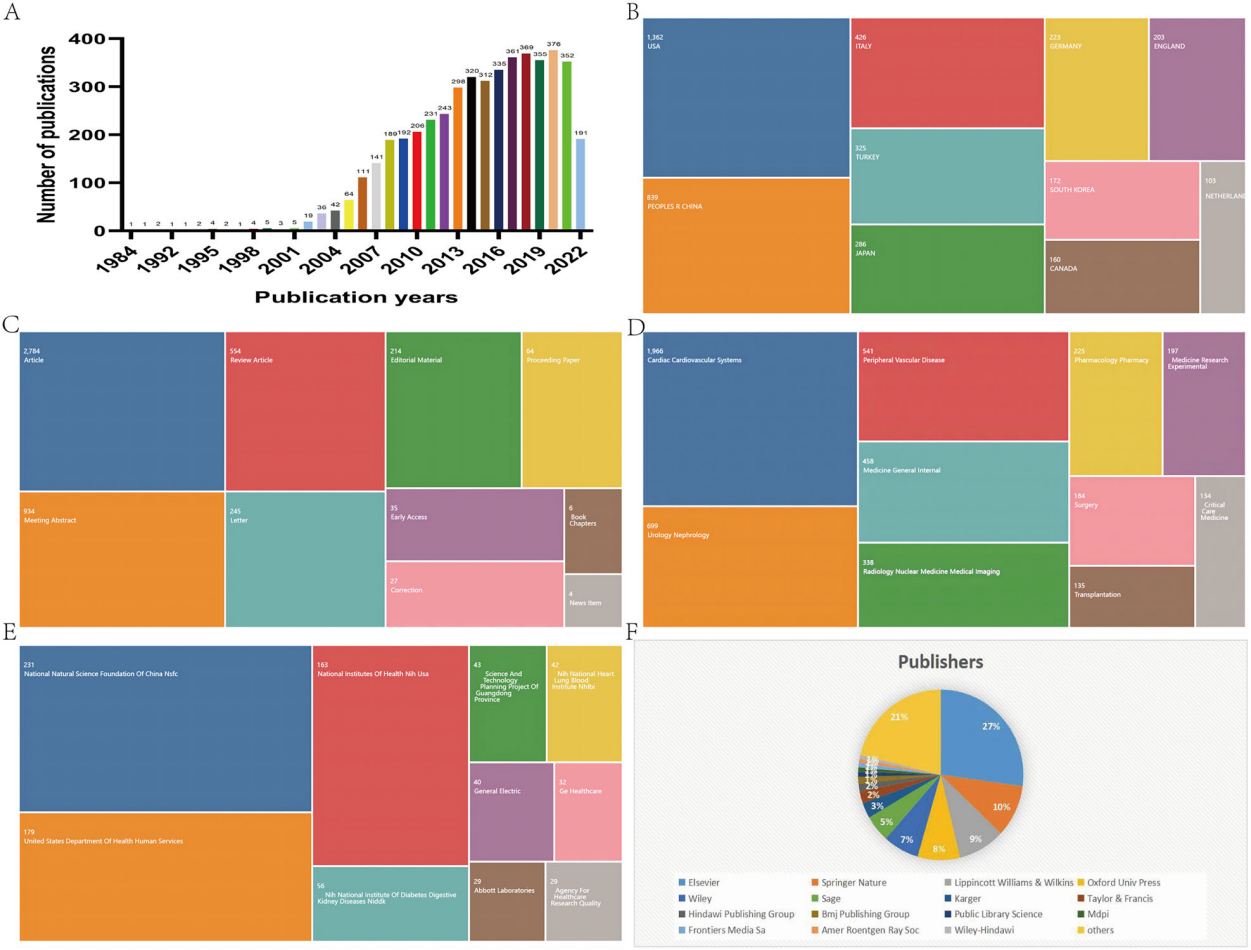
Global characteristics of articles published on CI-AKI. (A) Number of articles issued per year. (B) Number of articles published by country/region. (C) Type of article and corresponding number of publications. (D) WoS catalog classification of research areas. (E) Funding status of the article. (F) The publisher of the article.

We retrieved a total of 4775 articles from the WoSCC database. It has been 38 years since the concept of CI-AKI was first introduced in 1984, and the number of articles has shown a yearly growth and stabilization trend. Among them, the highest number of articles was published in 2020, with 376 articles, accounting for 7.874% (Figure 1(A)). These results suggest that CI-AKI research is attractive, and more prospective studies are still needed in the field of CI-AKI in the future.

From a global perspective, a total of 83 countries/regions published articles in this field. Among them, USA has the highest number of publications with 1362 articles, while PEOPLES R CHINA (839), ITALY (426), and TURKEY (325) are also countries with high publication volume in this field (Figure 1(B)).

#### 3.1.2. Article type

Dividing all the articles by type (Figure 1(C)), the highest percentage of these 4775 articles were monographs (2784, 58.304%), followed by conference abstracts (934, 19.560%), reviews (554, 11.602%), and letters (245, 5.131%).

#### 3.1.3. Research areas

In the WoS catalog display, a total of 88 research areas are specified (Figure 1(D)). Most of the literature is focused on ‘Cardiac Cardiovascular Systems’ (1966, 41.173%), ‘Urology Nephrology’ (699, 14.639%), ‘Peripheral Vascular Disease’ (541, 11.330%), ‘Medicine General Internal’ (458, 11.339%), and ‘Medicine General Internal’ (458, 11.343%). 14.639%), ‘Peripheral Vascular Disease’ (541, 11.330%), ‘Medicine General Internal’ (458, 9.592%), Radiology Nuclear Medicine. 9.592%) and Radiology Nuclear Medicine Medical Imaging (338, 7.079%). This demonstrates that CI-AKI is a multifaceted and multidisciplinary field of study covering a wide range of societal benefits.

#### 3.1.4. Fund support

Funding support in this area is shown in Figure 1(E), with the National Natural Science Foundation of China (231) providing the highest amount of financial support, followed by the United States Department of Health Human Services (179), National Institutes of Health (163), National Institute of Diabetes Digestive Kidney Diseases (56), Science and Technology Planning Project of Guangdong Province (43), and National Heart Lung Blood Institute (42). This indicates that the United States and China are the largest sources of financial support in this field.

#### 3.1.5. Publishers

Articles in this field were published by 260 publishers (Figure 1(F)). Of these, Elsevier (1296, 27.141%) was the largest publisher in terms of number of articles published, followed by Springer Nature (473, 9.906%), Lippincott Williams & Wilkins (439, 9.194%), Oxford Univ Press (395. 8.272%), and Wiley (336, 7.037%).

### 3.2. Visual analysis of countries/regions and institutions

**Figure 2.**
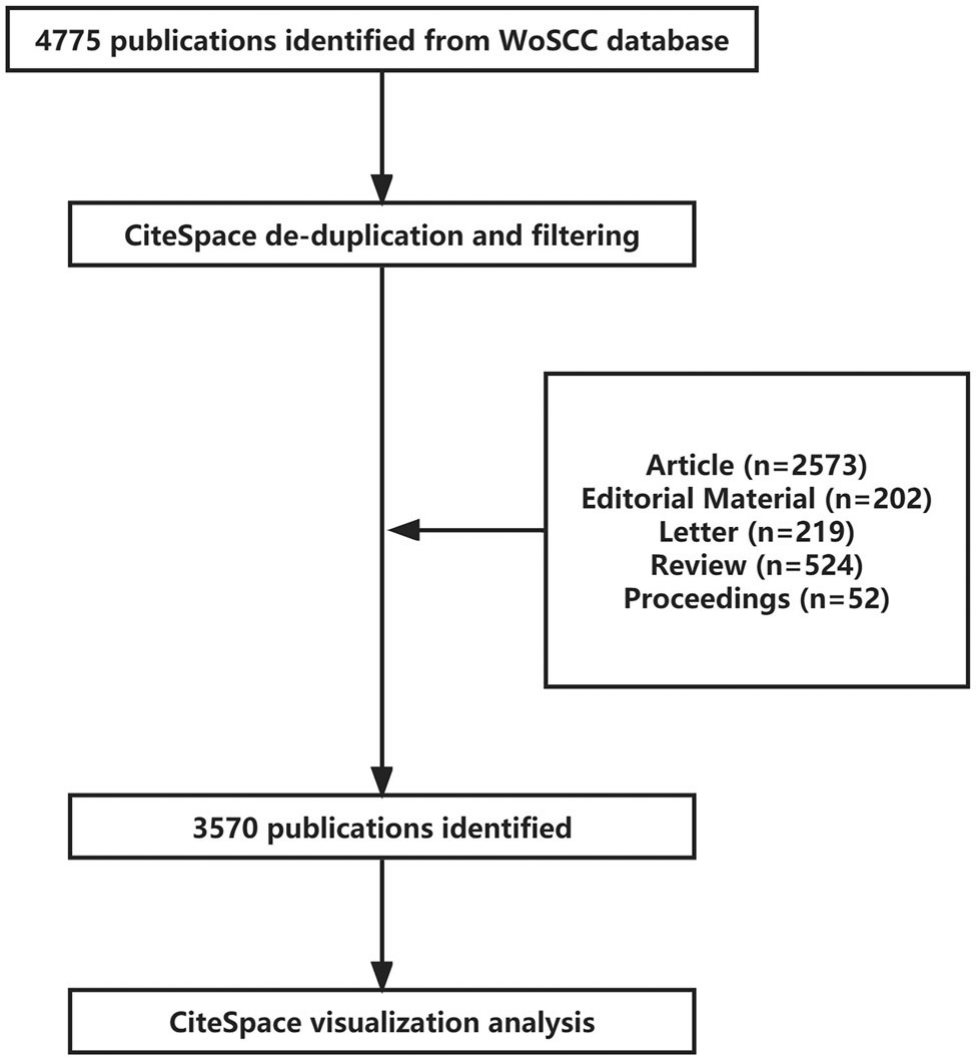
Flowchart of the screening process.

On 25 August 2022, we retrieved a total of 4775 articles through WoS, and after importing them into CiteSpace 6.1.R3 software, 3570 articles were extracted for analysis after data deduplication and cleaning. There were five types of articles: reviews, proceedings, editorial material, and letters. The 3570 articles were all in English, from 673 research institutions in 83 countries/regions, and published in 854 journals (an article could be counted multiple times because multiple countries/regions, institutions, and authors are involved). The screening process is shown in Figure 2.

#### 3.2.1. Countries/regions

**Figure 3.**
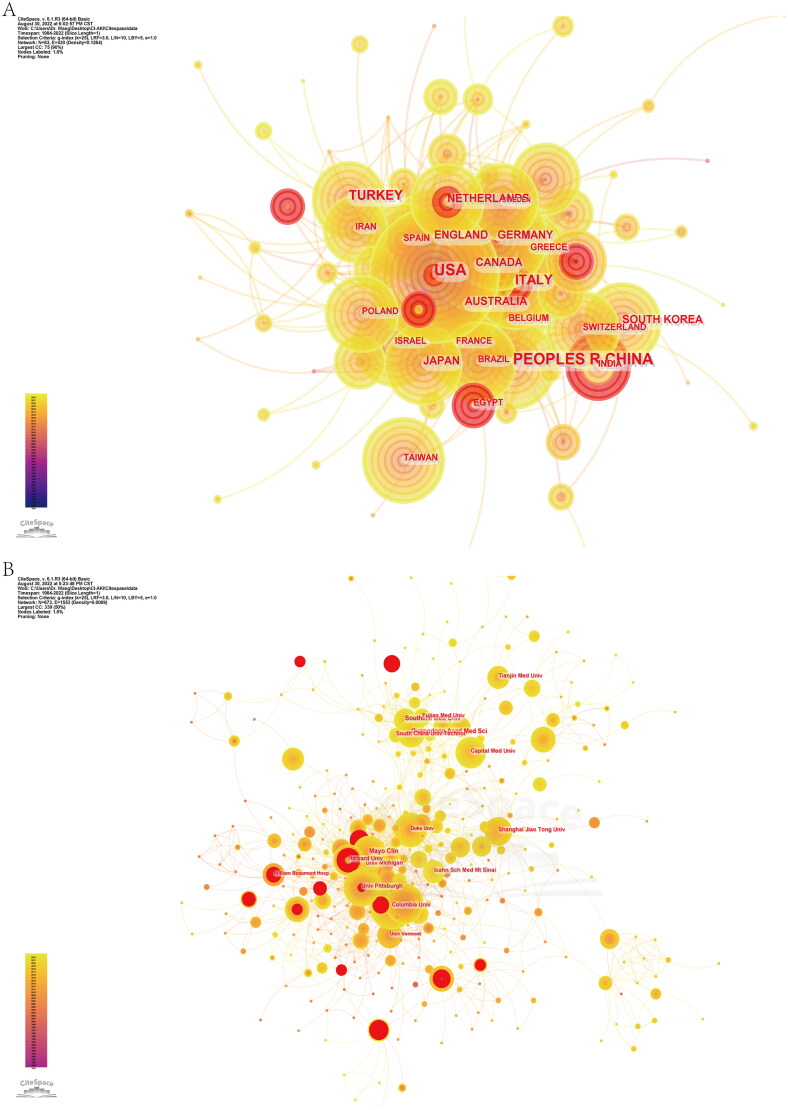
A network of national/regional and institutional cooperation in the field of CI-AKI. (A) The collaboration network of countries/regions. (B) The collaboration network of institutions.

Through CiteSpace analysis, the ten countries with the highest number of papers had a total article output of 73.45% (Figure 3(A), Table 1), with China and Turkey being the only developing countries. The top three countries/regions were the United States (1136, 24.54%), China (711, 15.36%), and Italy (330, 7.13%). Burst detection was used to detect sudden changes in topics, literature, authors, and journal citation information and may be the result of a breakthrough in the field. The USA was the country with the highest intensity and longest duration of citation bursts (15.22), followed by Germany (13.77), Canada (12.02), and Italy (7.52); Egypt, India, Pakistan, and Saudi Arabia were the countries with new citation bursts in the last three years (Supplementary Figure 1(A)). It is worth noting that China, the country publishing the second most articles, did not experience any citation burst. Centrality, also known as mediator centrality, is a measure whose higher values represent a more active and close role a node plays in cooperative relationships with other nodes. Individuals with high centrality are marked as purple circles in the graph. The United States had the highest centrality (0.56), followed by Germany (0.18), the United Kingdom (0.16), and Italy (0.15), indicating that they each play an important role as a bridge in the network of national cooperation in this field.

**Table 1.** The top 10 countries/regions and institutions involved in CI-AKI.

Rank	Country/region	Counts	Centrality	Citations	Institution	Counts	Centrality	Citations
1	USA	1136 (24.54%)	0.56	49,621	Guangdong Acad Med Sci	82 (2.47%)	0.02	838
2	CHINA	711 (15.36%)	0.04	8898	Southern Med Univ	82 (2.47%)	0.01	930
3	ITALY	330 (7.13%)	0.15	13,620	Mayo Clin	59 (1.78%)	0.07	6610
4	TURKEY	294 (6.35%)	0.07	3557	Univ Michigan	52 (1.57%)	0.06	7342
5	GERMANY	205 (4.43%)	0.18	10,791	Univ Pittsburgh	52 (1.57%)	0.04	5466
6	JAPAN	194 (4.19%)	0.01	3340	Columbia Univ	51 (1.54%)	0.03	7338
7	UK	176 (3.80%)	0.16	10,370	Fujian Med Univ	44 (1.33%)	0.02	192
8	CANADA	146 (3.15%)	0.05	7280	Shanghai Jiao Tong Univ	43 (1.30%)	0.05	1700
9	SOUTH KOREA	114 (2.46%)	0.03	1932	Tianjin Med Univ	40 (1.21%)	0.01	473
10	AUSTRALIA	94 (2.03%)	0.04	4751	South China Univ Technol	37 (1.12%)	0	194

According to VoSviewer’s analysis (Supplementary Figure 1(B)), the country/region with the highest number of citations was the United States (49,621), followed by Italy (13,620), Germany (10,791) and the United Kingdom (10,370). In addition, the connecting lines between nodes represent the cooperation between countries/regions, and the thickness of the connecting lines represents the intensity of cooperation. We found that many countries/regions cooperated extensively with each other, the most obvious cooperation being between China and the United States. The total link strength was significantly higher in the U.S. than in other countries, indicating that it works more closely with other countries.

#### 3.2.2. Institutions

According to the analysis of CiteSpace data (Figure 3(B), Table 1), the total number of submissions from the top 10 institutions accounted for 16.34% of the total number of manuscripts. The research institution collaboration network is made up of Guangdong Acad Med Sci (82, 2.47%), Southern Med Univ (82, 2.47%), Mayo Clin (59, 1.78%), Univ Michigan (52, 1.57%), Univ Pittsburgh (52, 1.57%) and several other research institutions form major research collaborations with the highest number of publications. Notably, six of the top ten institutions are from China: Guangdong Acad Med Sci, Southern Med Univ, Fujian Med Univ, Shanghai Jiao Tong Univ, Tianjin Med Univ, and South China Univ Technol. For citation burst strength detection (Supplementary Figure 1(C)), the top three were William Beaumont Hosp (13.67), Univ Michigan (10.42), and Harvard Univ (9.51), while Nanjing Med Univ (2017–2022) was an emerging research institution in recent years. In addition, several institutions, such as Duke Univ (0.13), Mayo Clin (0.07), Univ Michigan (0.06), Shanghai Jiao Tong Univ (0.05), and Icahn Sch Med Mt Sinai (0.05), showed high centrality, which implies that these institutions are important in the field of CI-AKI research.

According to the analysis of VoSviewer data (Supplementary Figure 1(D)), Univ Michigan (7342), Columbia Univ (7338), and Mayo Clin (6610) had the most total citations. Columbia Univ had the highest total link strength, followed by William Beaumont Hosp, Univ Michigan, and Univ Pittsburgh, indicating that U.S. institutions are more focused on communication and collaboration.

### 3.3. Visual analysis of journals and cocited journals

**Figure 4.**
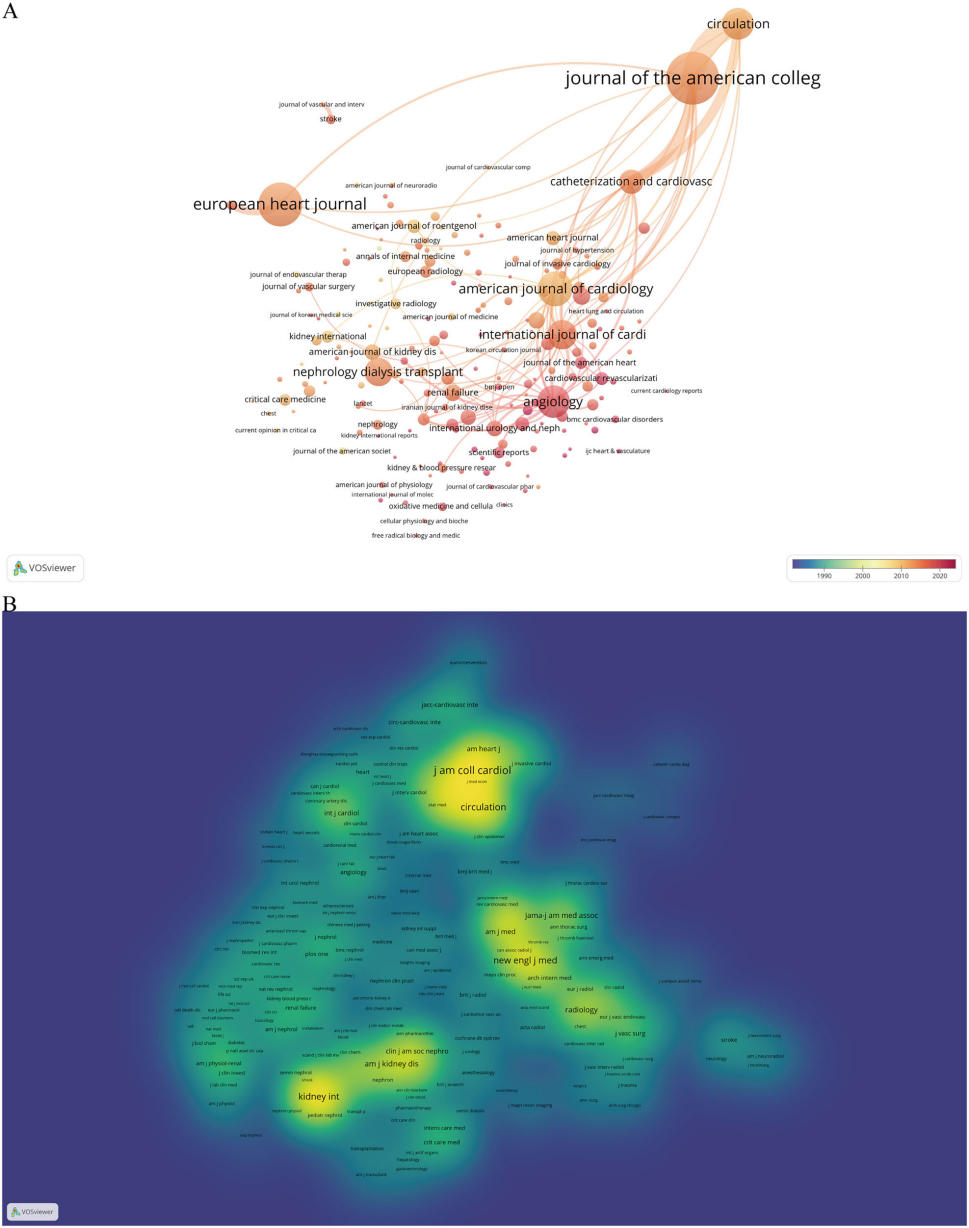
Visual analysis of journals. (A) The network of article source journals. (B) The network of cocited journals.

VoSviewer shows a total of 4775 articles related to the field of CI-AKI published in 854 journals. The top 10 journals with the most published articles and the 10 most cocited journals are listed in Table 2. Among these journals (Figure 4(A)), J AM COLL CARDIOL (297, IF = 27.203) published the most articles, followed by EUR HEART J (224, IF = 35.855), AM J CARDIOL (155, IF = 3.133) and ANGIOLOGY (141, IF = 3.299). Of these, seven of the top ten journals were in JCR Q2 and above. Among the cocited journals (Figure 4(B)), J AM COLL CARDIOL (9288, IF = 27.203) was the most cited, followed by CIRCULATION (6918, IF = 39.918), NEW ENGL J MED (6054, IF = 176.0774), and KIDNEY INT (5794, IF = 18.998). Nine of the top ten journals were in JCR Q1. This indicates the high quality and impact of journals related to the CI-AKI field.

**Table 2.** The top 10 journals and cocited journals related to CI-AKI.

Rank	Journal	Counts	IF (2021)	JCR (2021)	Co-cited journal	Citations	IF (2021)	JCR (2021)
1	J AM COLL CARDIOL	297	27.203	Q1	J AM COLL CARDIOL	9288	27.203	Q1
2	EUR HEART J	224	35.855	Q1	CIRCULATION	6918	39.918	Q1
3	AM J CARDIOL	155	3.133	Q3	NEW ENGL J MED	6054	176.0774	Q1
4	ANGIOLOGY	141	3.299	Q3	KIDNEY INT	5794	18.998	Q1
5	CIRCULATION	135	39.918	Q1	AM J CARDIOL	5562	3.133	Q3
6	INT J CARDIOL	119	4.039	Q2	AM J KIDNEY DIS	3481	11.072	Q1
7	NEPHROL DIAL TRANSPL	112	7.186	Q1	J AM SOC NEPHROL	3431	14.978	Q1
8	CATHETER CARDIO INTE	90	2.585	Q3	RADIOLOGY	3277	29.146	Q1
9	JACC-CARDIOVASC INTE	53	11.075	Q1	JAMA-J AM MED ASSOC	3100	157.335	Q1
10	HEART	48	7.365	Q1	NEPHROL DIAL TRANSPL	2935	7.186	Q1

**Figure 5.**
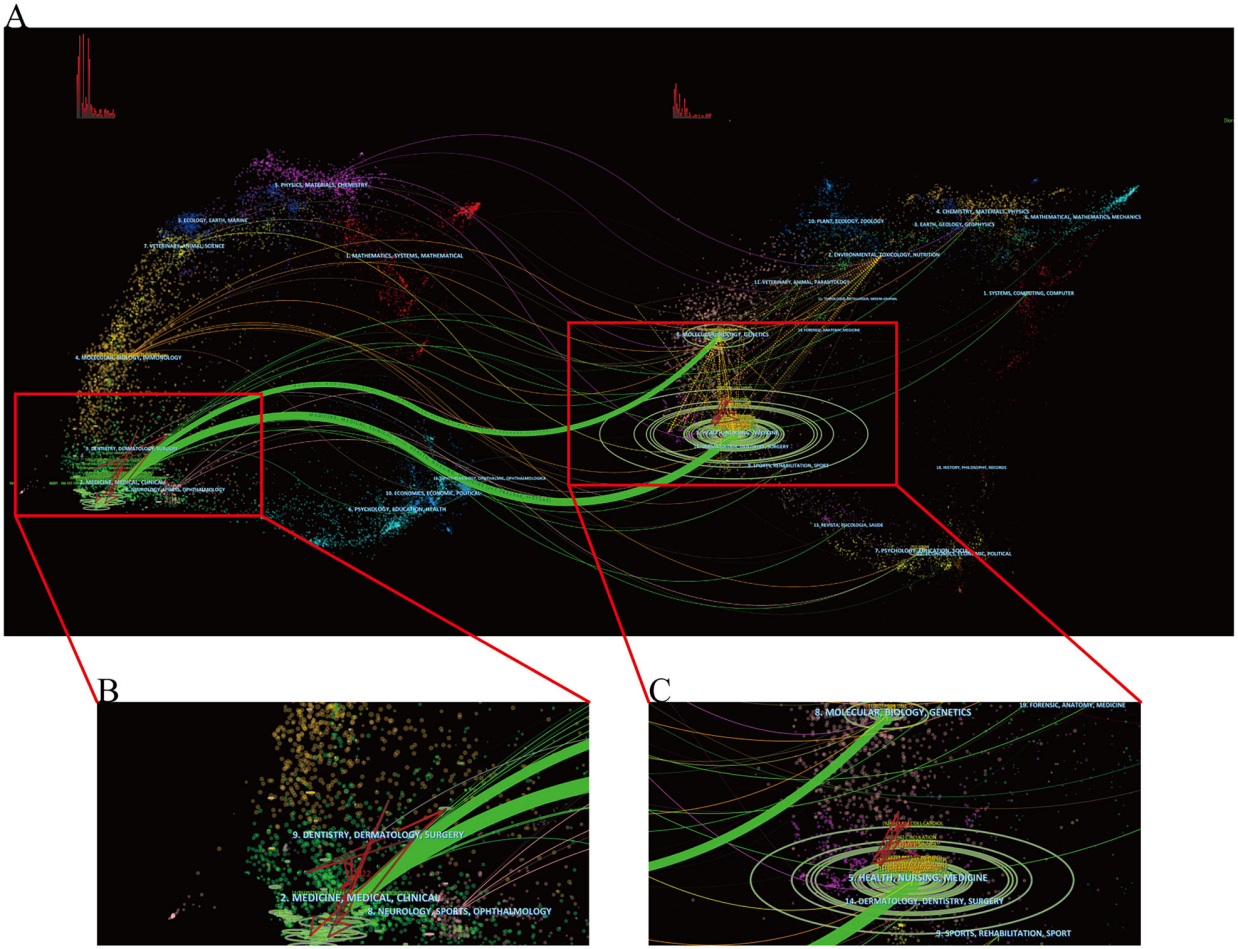
The dual-map overlay of journals.

The dual-map overlay of journals is a new way to display information about the distribution, citation trajectories, and drift of gravity of papers across disciplines. In Figure 5, the left side is the citing journal, the right side is the cited journal, and the curve is the citation linkage, which shows the complete citation relationship. In the dual-map overlay of journals, the more articles a journal publishes, the longer the vertical axis of the ellipse; the more authors it has, the longer the horizontal axis of the ellipse. Two of the main reference paths (in green) indicate that articles published in molecular/biology/genetics and health/nursing/medicine journals are frequently cited by medicine/medical/clinical journals.

### 3.4. Visual analysis of authors and cocited authors

**Figure 6.**
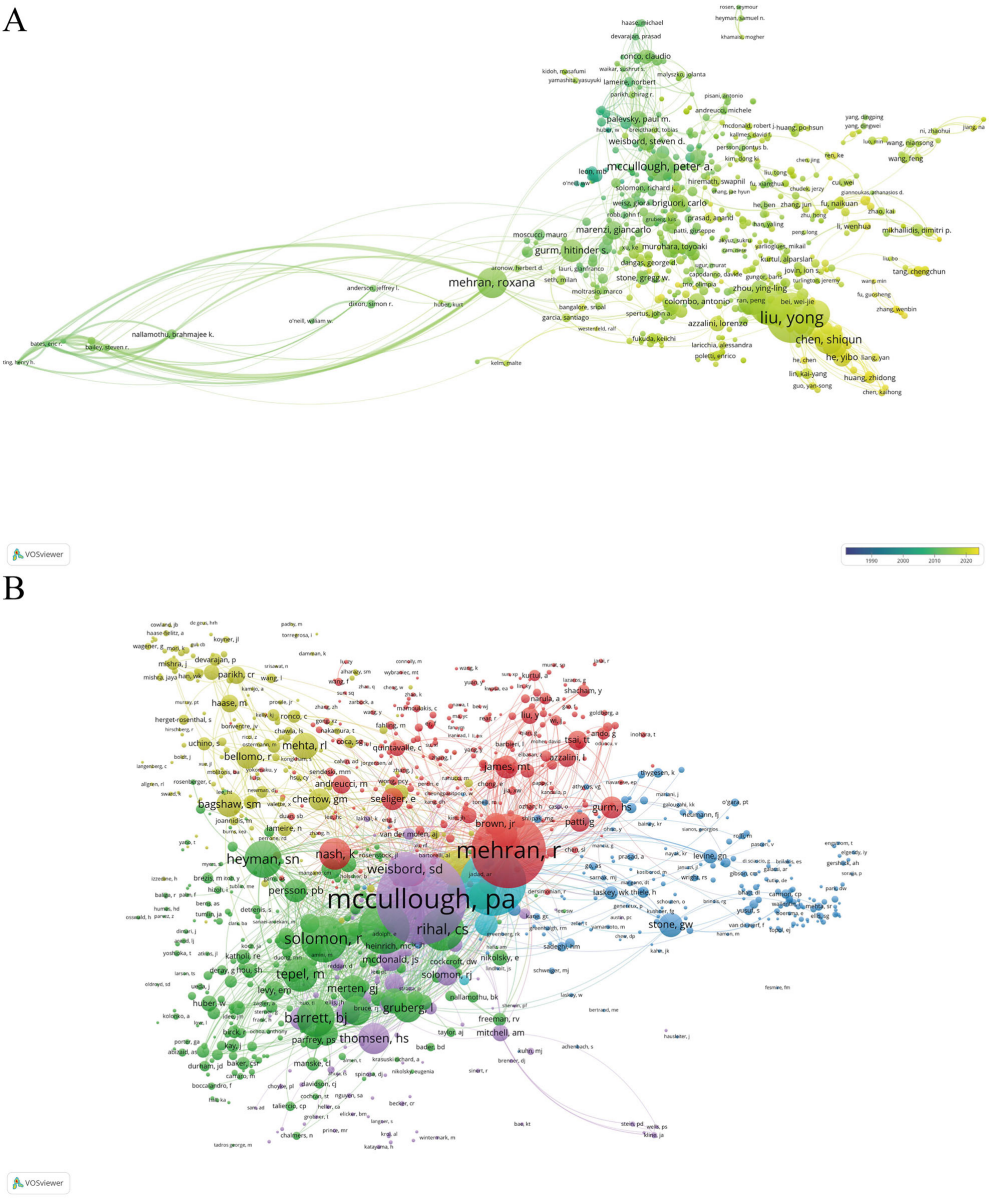
Visual analysis of authors. (A) The collaboration network of scholars. (B) The network of cocited authors.

CiteSpace showed that a total of 17,357 authors were involved in the publication of these articles (Figure 6(A), Table 3). Liu Y was an author on the most articles (147), followed by Chen J (111), Tan N (94), and Chen S (81). We also note the total number of citations of the authors, as it reflects the recognition of their results in the field. Mehran R (8257) was the author with the most total citations, publishing 77 articles; Blankenship Jc published only nine articles but ranked second in total citations, while authors with the highest number of publications, such as Liu Y (1258 citations), did not have significant total citations. In addition, Mehran R ranked first in total link strength, indicating that he collaborated closely with other researchers.

**Table 3.** The top 10 authors and cocited authors of CI-AKI research.

Rank	Author	Counts	Citations	Total link strength	Co-cited author	Citations	Total link strength
1	Liu, Y	147	1258	3752.63	Mccullough, PA	2316	2221.96
2	Chen, J	111	756	2904.12	Mehran, R	1773	1732.42
3	Tan, N	94	781	2178.73	Marenzi, G	1495	1414.22
4	Chen, S	81	384	2093.39	Briguori, C	1052	1024.21
5	Mehran, R	77	8257	3943.29	Solomon, R	925	907.02
6	Liu, J	64	250	1611.97	Rihal, CS	827	825.56
7	Mccullough, Pa	56	3525	2163.94	Barrett, BJ	695	680.33
8	Li, H	55	433	1452.4	Heyman, SN	683	652.55
9	Zhang, J	52	568	1429.84	Weisbord, SD	633	621.9
10	Li, Y	49	599	1434.82	Levey, AS	632	619.22

VoSviewer showed that the total number of cocited authors was 39,940 (Figure 6(B), Table 3). Cocited author analysis refers to two articles by two authors being cited by a third article. The higher the number of cocitations, the closer the researcher’s academic interests and the greater the research density. Sixty-two authors had more than 200 cocitations in this study, and the most frequently cocited author was Mccullough Pa (2316), followed by Mehran R (1773), Marenzi G (1495), and Briguori C (1052).

### 3.5. Visual analysis of articles and cocited references

Citation analysis was performed on the retrieved literature (Supplementary Figure 2(A)), and VoSviewer showed 203 articles with total citations above 100, and the 10 articles with the highest total citations are shown in Table 4. Nine of the articles were in the review or guideline recommendation category, and one was a clinical study. The article titled ‘KDIGO clinical practice guidelines for acute kidney injury’ had the highest total citations, but the link strength was low, indicating a weak linkage to other articles in the CI-AKI field.

**Table 4.** The top 10 articles on CI-AKI.

Rank	Year	First author	Title	Total citations	Total link strength
1	2012	Khwaja [29]	KDIGO clinical practice guidelines for acute kidney injury	2134	4
2	2011	Levine [30]	2011 ACCF/AHA/SCAI Guideline for Percutaneous Coronary Intervention: A Report of the American College of Cardiology Foundation/American Heart Association Task Force on Practice Guidelines and the Society for Cardiovascular Angiography and Interventions	2131	24
3	2004	Mehran [31]	A simple risk score for prediction of contrast-induced nephropathy after percutaneous coronary intervention: Development and initial validation	1633	65
4	2018	Aboyans [32]	2017 ESC Guidelines on the Diagnosis and Treatment of Peripheral Arterial Diseases, in collaboration with the European Society for Vascular Surgery (ESVS)	1138	1
5	2013	Kellum [33]	Diagnosis, evaluation, and management of acute kidney injury: a KDIGO summary (Part 1)	1102	3
6	2009	Kushner [34]	2009 Focused Updates: ACC/AHA Guidelines for the Management of Patients With ST-Elevation Myocardial Infarction (updating the 2004 Guideline and 2007 Focused Update) and ACC/AHA/SCAI Guidelines on Percutaneous Coronary Intervention (updating the 2005 Guideline and 2007 Focused Update): a report of the American College of Cardiology Foundation/American Heart Association Task Force on Practice Guidelines	1076	6
7	2009	Kushner [35]	2009 Focused Updates: ACC/AHA Guidelines for the Management of Patients With ST-Elevation Myocardial Infarction (Updating the 2004 Guideline and 2007 Focused Update) and ACC/AHA/SCAI Guidelines on Percutaneous Coronary Intervention (Updating the 2005 Guideline and 2007 Focused Update): A Report of the American College of Cardiology Foundation/American Heart Association Task Force on Practice Guidelines	1076	16
8	2007	Levin [36]	KDOQI Clinical Practice Guidelines and Clinical Practice Recommendations for Diabetes and Chronic Kidney Disease	969	4
9	2011	Levine [30]	2011 ACCF/AHA/SCAI Guideline for Percutaneous Coronary Intervention: a report of the American College of Cardiology Foundation/American Heart Association Task Force on Practice Guidelines and the Society for Cardiovascular Angiography and Interventions	953	26
10	2012	Bellomo [37]	Acute kidney injury	923	9

When two articles are cited by a third article at the same time, it is called a literature cocitation relationship. References are often considered the knowledge base of a specific field, and the more frequently a paper is cited, the more significant it is in a particular field. Based on the number of cocitations, we selected the top ten references, each of which was cited at least 120 times in the CI-AKI field (Table 5). Of the top 10 cocited articles, five were from the United States and three were from Italy; seven were clinical trials and three were reviews. The most cocited clinical study, entitled ‘Prevention of contrast-induced nephropathy with sodium bicarbonate: a randomized controlled trial’ (170), was published by Merten et al. [38] in the Journal of the American Medical Association. They concluded that hydration with sodium bicarbonate before contrast injection was more effective in preventing contrast-induced renal failure than hydration with sodium chloride. A review entitled ‘Contrast-Associated Acute Kidney Injury’ (126), published in The New England Journal of Medicine, provides more detailed information on the pathophysiology, diagnostic criteria, and risk stratification and prevention of CI-AKI [44]. An article published in the journal Annals of Internal Medicine entitled ‘Meta-analysis: effectiveness of drugs for preventing contrast-induced nephropathy’ had the greatest centrality (0.19), indicating a significant impact on other researchers [47].

**Table 5.** The top 10 cocited references of CI-AKI research.

Rank	Year	First author	Title	Local citations	Centrality
1	2004	Merten GJ [38]	Prevention of contrast-induced nephropathy with sodium bicarbonate: a randomized controlled trial	170	0.09
2	2017	Nijssen EC [39]	Prophylactic hydration to protect renal function from intravascular iodinated contrast material in patients at high risk of contrast-induced nephropathy (AMACING): a prospective, randomized, phase 3, controlled, open-label, non-inferiority trial	152	0.04
3	2007	Briguori C [40]	Renal Insufficiency Following Contrast Media Administration Trial (REMEDIAL): a randomized comparison of 3 preventive strategies	139	0.02
4	2008	Mccullough PA [41]	Contrast-induced acute kidney injury	137	0.03
5	2006	Marenzi G [42]	N-Acetylcysteine and Contrast-Induced Nephropathy in Primary Angioplasty	133	0.04
6	2018	Weisbord SD [43]	Outcomes after Angiography with Sodium Bicarbonate and Acetylcysteine	132	0.04
7	2019	Mehran R [44]	Contrast-Associated Acute Kidney Injury	126	0.03
8	2003	Aspelin P [45]	Nephrotoxic effects in high-risk patients undergoing angiography	125	0.07
9	2011	Stacul F [46]	Contrast induced nephropathy: updated ESUR Contrast Media Safety Committee guidelines	121	0.01
10	2004	Mehran R [31]	A simple risk score for prediction of contrast-induced nephropathy after percutaneous coronary intervention: development and initial validation	120	0.06

A citation burst in the cocited literature is a sudden increase in guideline articles over a certain period of time. The 13 most intense bursts of literature were obtained through CiteSpace (Supplementary Figure 2(B)), with the selection criterion of a minimum duration of 6 years. The most bursty reference was Merten’s article, which also had the highest number of cocitations. In addition, an article entitled ‘Effectiveness of Prevention Strategies for Contrast-Induced Nephropathy: A Systematic Review and Meta-analysis’ [48] was published in the Annals of Internal Medicine journal and is currently a popular article with a burst from 2016 to 2022.

**Figure 7.**
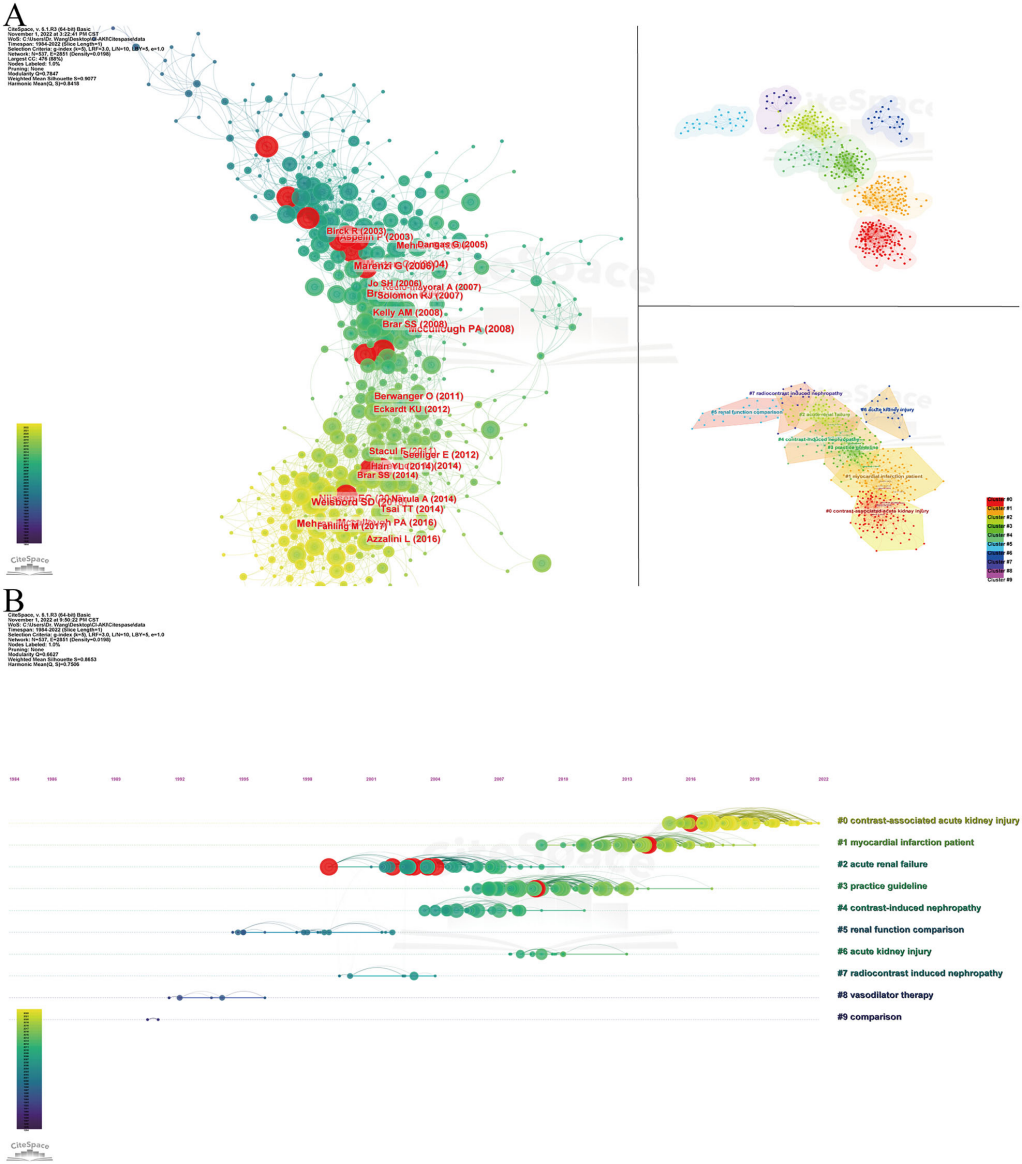
Visual analysis of cocited references. (A) Cluster analysis of cocited references. (B) The clustering timeline map of cocited references.

We used CiteSpace software to generate reference cocitation maps with corresponding clusters so that significant references and research clusters can be extracted. A total of 36 clusters were formed using the LLR algorithm, and we selected the top 10 clusters with significant modularity and profile scores (*Q* = 0.8645, *S* = 0.8964) (Figure 7(A), Table 6). *Q* values range from 0 to 1, and values >0.3 indicate a clear circling structure. *S* values >0.5 indicate reasonable clustering, and values >0.7 are more accurate. According to the LLR algorithm, the top ten clusters are contrast-associated acute kidney injury (cluster #0), contrast-associated acute kidney injury (cluster #1), acute renal failure (cluster #2), practice guideline (cluster #3), contrast-induced nephropathy (cluster #4), renal function comparison (cluster #5), acute kidney injury (cluster #6), radiocontrast-induced nephropathy (cluster #7), vasodilator therapy (cluster #8), and comparison (cluster #9).

**Table 6.** The top 10 clusters of cocited references.

Cluster ID	Size	Silhouette	Label (LSI)	Label (LLR)	Label (MI)	Average year
0	118	0.935	Contrast-induced acute kidney injury	Contrast-associated acute kidney injury	Dehydration	2019
1	112	0.815	Contrast-induced nephropathy	Contrast-associated acute kidney injury	Dehydration	2014
2	79	0.881	Contrast-induced nephropathy	Acute renal failure	Dehydration	2004
3	71	0.88	Contrast-induced nephropathy	Practice guideline	Dehydration	2010
4	28	0.891	Contrast-induced nephropathy	Contrast-induced nephropathy	Pulmonary embolism	2006
5	27	0.968	Contrast-induced nephropathy	Renal function comparison	Contrast-induced nephropathy	1997
6	22	0.972	Acute kidney injury	Acute kidney injury	Contrast-induced nephropathy	2009
7	15	0.969	Renal function	Radiocontrast induced nephropathy	Contrast-induced nephropathy	2001
8	10	1	Contrast-induced nephrotoxicity—the effects of vasodilator therapy	Vasodilator therapy	Contrast-induced nephropathy	1993
9	8	1	Radiocontrast-associated renal dysfunction—a comparison of lower-osmolality and conventional high-osmolality contrast-media	Comparison	Contrast-induced nephropathy	1991

To reveal research trends and hotspots over time, we drew a timeline view of the cocited literature (Figure 7(B)). It was clear that cluster #0 was made up of by far the most popular cocited literature; cluster #2 contained the most literature with higher-level bursts; cluster #3 lasted the longest; and clusters #8 and #9 were unlinked to the other clusters, being relatively isolated.

### 3.6. Visual analysis of keywords

**Figure 8.**
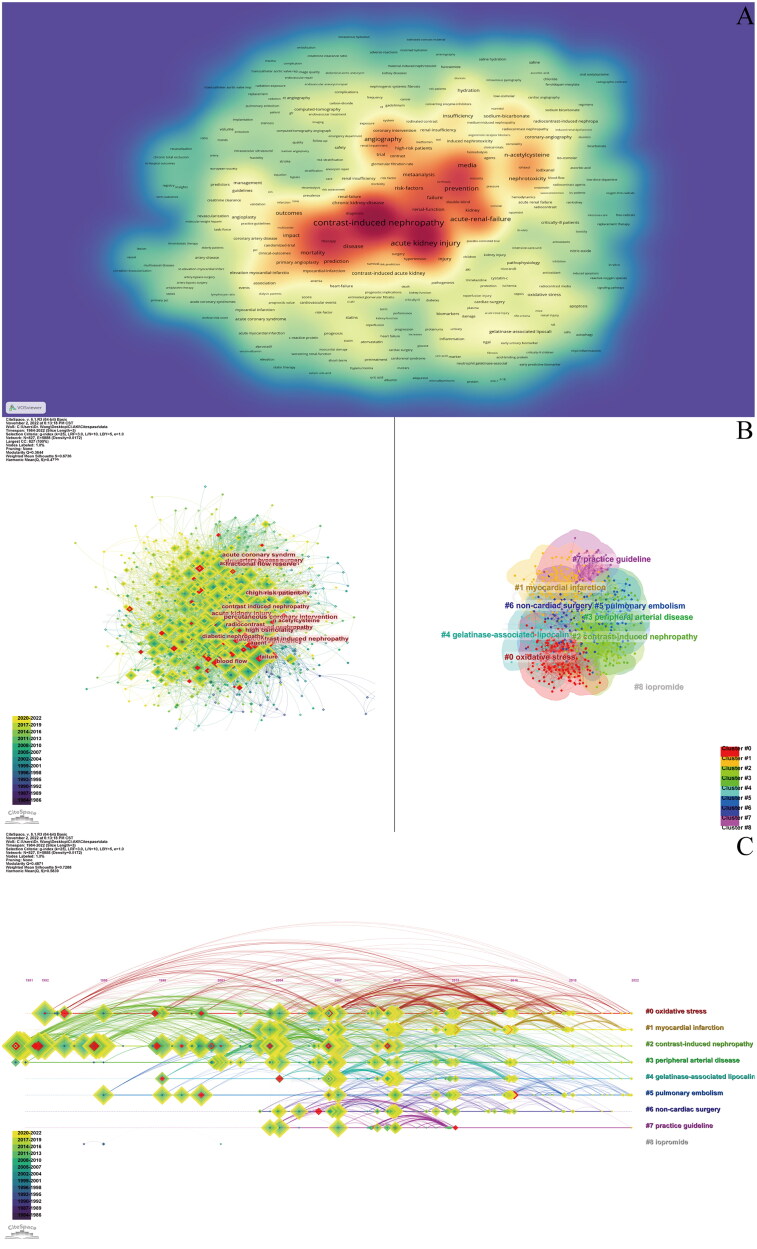
Visual analysis of keywords. (A) Heat map of keywords. (B) Cluster analysis of keywords. (C) The clustering timeline map of keywords.

Keywords usually reflect the topic and research content of the article. By analyzing the co-occurrence of keywords, we can quickly understand the research focus and research direction in the field of CI-AKI. We used CiteSpace and VOSviewer to construct a keyword co-occurrence network (Figure 8(A,B)). A total of 827 keywords were extracted, among which 150 keywords appeared 30 times or more. Table 7 shows the top 20 keywords. The most frequent keyword was ‘contrast-induced nephropathy’ (*n* = 1151), while the keywords with the highest centrality (0.04) were ‘computed tomography’, ‘high-risk patient’, ‘radiocontrast’, ‘abdominal aortic aneurysm’, ‘blood flow’, ‘high osmolality’, ‘diabetic nephropathy’, and ‘fractional flow reserve’. Excluding the conceptual terms related to CI-AKI and some meaningless words, ‘percutaneous coronary intervention’, ‘acute renal failure’, ‘angiography’, ‘chronic kidney disease’, ‘acetylcysteine coronary angiography’, ‘sodium bicarbonate’, ‘serum creatinine’, and other key words appeared more frequently. This suggests that most of the research on CI-AKI has focused on clinical trials related to risk factors for its onset, high-risk groups, and clinical presentation.

**Table 7.** The top 20 keywords of CI-AKI research.

Rank	Keywords	Centrality	Counts	Year
1	contrast induced nephropathy	0.03	1151	2002
2	acute kidney injury	0.03	1097	2007
3	percutaneous coronary intervention	0.02	966	2003
4	acute renal failure	0.02	776	1992
5	contrast-induced nephropathy	0	752	2005
6	induced nephropathy	0.02	731	1994
7	risk	0.01	631	1991
8	prevention	0.01	603	2000
9	media	0.01	525	1991
10	angiography	0	456	1994
11	risk factor	0.01	435	1992
12	chronic kidney disease	0.01	392	2005
13	contrast media	0.01	380	1993
14	n acetylcysteine	0.01	376	2002
15	disease	0.01	373	2002
16	outcome	0.01	367	2004
17	coronary angiography	0.02	362	2004
18	mortality	0.01	333	2003
19	intervention	0.01	300	2003
20	impact	0	286	2005

The keywords were clustered by CiteSpace’s LLR algorithm. Figure 8(B) shows that there are 9 clusters, namely, ‘oxidative stress’ (cluster #0), ‘myocardial infarction’ (cluster #1), ‘contrast-induced nephropathy’ (cluster #2), ‘peripheral arterial disease’ (cluster #3), ‘gelatinase-associated lipocalin’ (cluster #4), ‘pulmonary embolism’ (cluster #5), ‘non-cardiac surgery’ (cluster #6), ‘practice guideline’ (cluster #7), and ‘iopromide’ (cluster #8). The keywords mainly suggested the research directions of injury mechanism, etiology, risk factors, and practice guidelines of CI-AKI. The timeline view shows the evolution of the keywords, allowing you to visualize the keywords in the CI-AKI domain over time. As shown in Figure 8(C), cluster #1 is a relatively popular research direction at present; cluster #2 contains more highly emergent keywords, which are significant in the early research of CI-AKI; and clusters #7 and #8 are no longer a research hotspot.

A total of 73 keywords were analyzed among the citation bursts with a duration of 5 years (Supplementary Figure 3). The keyword with the highest burst was ‘randomized controlled trial’ (37.77). The citations with the longest burst durations were ‘nephrotoxicity’ and ‘clinical trial’ (24). The keywords with the longest citation burst durations were ‘elevation myocardial infarction’, ‘multivessel disease’, and ‘high risk’, representing the current hot research topics.

## 4. Discussion

### 4.1. CI-AKI global research quality

This paper is the first to use a bibliometric approach to provide a detailed and comprehensive visual analysis of existing articles in the field of CI-AKI research to provide guidance for future research. Based on our selection criteria, 4775 CI-AKI-related articles were obtained from the WoSCC database, spanning the years 1984–2022. Using CiteSpace software to deduplicate and clean the data, a total of 3570 articles were extracted from 673 research institutions in 83 countries/regions, published in a total of 854 journals. Five types of literature are included: articles, reviews, proceedings, editorial material, and letters. During this nearly 40-year period, the number of publications has been increasing and remains high, indicating rapid growth and continued research interest in the field of CI-AKI. We found that the number of articles exceeded 100 in 2006, 200 in 2010, and 300 in 2014. The sharp rise in the number of articles during this period may be related to the publication of various guides [49–56]. These guideline recommendations and expert consensuses provide guidance for clinical trials and establish diagnostic criteria for CI-AKI that can be used in animal studies.

Analysis of country/regional and institutional distribution helps to facilitate teamwork and global collaboration in a given field. The United States is the clear leader in CI-AKI, with the highest volume of publications, the highest intermediary centrality, and the highest intensity of citation bursts. This may be due to important contributions from research institutions, such as Mayo Clin, Univ Michigan, and Univ Pittsburgh. It is worth noting that the author with the highest total citations, Mehran R, and the most frequently cocited author, Mccullough Pa, are both from the United States. In contrast, China, the second largest country in terms of paper output, has the top two institutions and the top three authors in terms of publication volume. Unfortunately, the centrality of China and the global citations of its articles are low, and there has been no citation explosion. This suggests that China should focus on the quality and global impact of its articles.

The analysis of journals and cocited journals can help researchers to choose the right journal to submit their manuscripts. Among the top ten journals in terms of number of publications, JCR Q2 and above accounted for seven journals, and the journals with the largest number of publications and the highest IF were J AM COLL CARDIOL (297, IF = 27.203). Among the cocited journals, J AM COLL CARDIOL (9288, IF = 27.203) was the most frequently cited. Among the top ten journals in terms of cocitation frequency, the journal with the highest IF was NEW ENGL J MED (6054, IF = 176.0774), and nine journals were in JCR Q1. These data indicate that journals related to CI-AKI research have a high impact and are favored. In fact, of the top ten journals in terms of number of articles published in the field of CI-AKI research, only one is in the field of nephrology, and the others are in the cardiovascular and peripheral vascular fields. This suggests that CI-AKI-related research is more likely to be favored by specialists in the field of circulatory systems.

### 4.2. Current status of CI-AKI research

The main feature of CI-AKI is a decrease in renal function within a few days after intravascular administration of iodine contrast. The definition and diagnostic criteria of CI-AKI have evolved over time. Early on, CI-AKI was referred to as contrast-induced nephropathy (CIN), defined as acute impairment of renal function following exposure to CM, for which other etiologies have been ruled out [57]. Serial measurement of serum creatinine is considered the most practical and sensitive method, and an elevation of 1.0 mg/dL compared to baseline is an accepted diagnostic criterion [58]. In 2012, the KDIGO organization continued the name of CIN with the diagnostic criteria of renal impairment (elevation of serum creatinine above 25% or 44 μmol/L) within 3 days after intravascular contrast injection in the absence of other etiologies [5]. In 2018, ESUR recommended replacing ‘CIN’ with ‘postcontrast acute kidney injury’ (PC-AKI) and retaining the term ‘CI-AKI’ to describe the causal relationship between CM and deteriorating renal function [59]. The latest diagnostic criteria for CI-AKI are an increase in serum creatinine ≥0.3 mg/dL or ≥1.5–1.9 times the baseline level within 48–72 h after administration of CM. This guideline is also followed by the French Nephrology societies (SFNDT) and the French Radiological Society (SFR) [60]. Given the clinical value of CI-AKI, guidelines from the clinical departments of nephrology, cardiovascular medicine, and peripheral vasculature are even more important. Notably, a consensus from the American College of Radiology and the National Kidney Foundation pointed out the difference between CA-AKI and CI-AKI [61]. Due to the historical confusion between CA-AKI and CI-AKI, many articles do not make the distinction [62]. CA-AKI is defined as any AKI that occurs within 48 h of the administration of contrast media [63]. It is clear that CI-AKI should be a subset of CA-AKI, as CI-AKI implies a causal relationship between contrast injection and AKI. In clinical practice, however, this correlation *vs.* causation is not well distinguished, leading to a possible exaggeration of the harm caused by contrast media [61]. It may be that this potential causal relationship can only be established if well-matched controls are included in a study. There is a paucity of this type of article.

The incidence of CI-AKI is related to the contrast agent, the patient’s own condition, and the type of procedure. The influence of the contrast agent itself on the incidence of CI-AKI is mainly reflected in cocited literature cluster #9. Lautin et al. [64] found that hypotonic CM reduced CI-AKI to varying degrees compared to hyperosmotic CM. In contrast, hypotonic CM is more toxic than isotonic CM and more likely to cause endothelial dysfunction and disorders of the prothrombin and fibrinolytic systems in *ex vivo* experiments in rats [65]. In the cocited literature, cluster #4 mainly shows the relevance of underlying diseases for the occurrence of CI-AKI. For example, CKD, diabetes mellitus, congestive heart failure, hypotension, peripheral vascular disease, anemia, and age are all high-risk factors for CI-AKI [31,66,67].

Interestingly, the risk of developing CI-AKI appears to differ by route of administration. In some retrospective studies, CI-AKI caused by intravenous and intra-arterial iodixanol injections was not significantly different and was no more common than after unenhanced CT scans [68–70]. However, in a prospective study of patients with suspected coronary artery disease, intravenous contrast for coronary CT imaging produced a lower rate of CI-AKI than arterial contrast for conventional coronary angiography [71]. The intravenous route of administration reduces the concentration of contrast agent delivered to the kidney and avoids microcirculatory embolism from the aorta to the kidney compared to intra-arterial injection [44,72]. For example, patients with ST-segment elevation infarction who undergo PCI are at higher risk of CI-AKI [73]. Wang et al. found that the same dose of contrast agent injected through the renal artery was more significant than that injected through the femoral artery or ear vein in a rabbit CI-AKI model [74].

Cocited literature clusters #0 and #2 are mainly concerned with clinical prevention strategies for CI-AKI. Currently, prophylactic hydration remains an important strategy to protect renal function from intravascular iodine CM, and sodium bicarbonate, saline, N-acetylcysteine, and ascorbic acid are commonly used clinical hydration agents [38–40,42,43]. In addition, statins may also prevent the development of CI-AKI [75–77]. Interestingly, in cluster #8, it was noted that vasodilator therapy with dobutamine inhibited the occurrence of CI-AKI [78].

The pathophysiological mechanisms of CI-AKI are not fully understood, but direct and indirect cytotoxic effects and hemodynamic alterations remain the main mechanisms of injury [44]. In keyword clusters #0 and #4, the keywords ‘oxidative stress’ and ‘nitric oxide’ were associated with pathophysiological mechanisms, while ‘cystatin c’ and ‘gelatinase-associated lipocalin’ were relevant markers of injury. Direct toxic effects of CM on renal tubular epithelial cells lead to cell death and dysfunction [22,79]. Indirect effects are mainly related to ischemic-hypoxic injury due to vasodilatory changes mediated by vasoactive substances, such as endothelin, reactive oxygen species, nitric oxide, and prostaglandins [80–83]. The outer renal medulla has a relatively low partial pressure of oxygen, which when combined with enhanced metabolic demands makes the medulla particularly susceptible to the hemodynamic effects of CM [83–85]. In addition, we found that the pathological changes in the peritubular capillaries precede those in the renal tubules in the early stage of CI-AKI, and the related molecular mechanisms are being investigated.

### 4.3. Limitations

In this study, we used CiteSpace and VOSviewer software for visual analysis of the WoSS database and may have missed papers published in other databases. Since the search strategy we created was designed to obtain the most comprehensive data possible, the topics of the included articles may not be highly relevant to CI-AKI. In addition, both software programs cannot obtain information on all authors of articles, cannot distinguish the order of authors, cannot combine different formats of the same author name, and may not evaluate the roles played by researchers in the field with sufficient precision.

### 4.4. Future vision of CI-AKI

Currently, most of the research on CI-AKI is done in clinical trials, while animal models also play an important role in the study of the molecular mechanisms of CI-AKI. Clinical trials play an important role in investigating risk factors, prevention strategies, and treatment options for CI-AKI and should be a hot spot for future research. We believe that research on CI-AKI should adopt a multicentric, multi-institutional model, increase the sample sizes, do longer patient follow-up, and collect the most comprehensive clinical data and laboratory and imaging data possible. In addition, there is a paucity of basic research on clinical specimens. Multiomics analysis of human specimens or single-cell sequencing should be performed in compliance with ethical requirements, which is expected to yield more information on the mechanism of injury.

## 5. Conclusion

Based on a visual grid analysis built with CiteSpace and VOSviewer software, we discuss trends emerging from collaborative networks, key clustering, citation bursts, and time-line relationships. Through bibliometric analysis, we find that the main areas of knowledge in the field of CI-AKI research are risk factors for CI-AKI, prevention and treatment strategies, evaluation of the effects of CM, and potential mechanisms of injury. From the keywords and cocited literature, clinical trials are still a popular direction for CI-AKI research today. The progress of research in the field of CI-AKI is explored through a visual analysis of the literature using a bibliometric approach. This method can help researchers visualize the current state of research and future trends. Compared to traditional reviews, bibliometrics has its own unique value but also suffers from a lack of research depth. We are grateful to the development team of CiteSpace and VOSviewer software and hope that they will further optimize the software features to achieve a more accurate and in-depth visual analysis of the research field.

## Supplementary Material

Supplemental Material

Supplemental Material

Supplemental Material

## References

[1] Tao SM, Wichmann JL, Schoepf UJ, et al. Contrast-induced nephropathy in CT: incidence, risk factors and strategies for prevention. Eur Radiol. 2016;26(9):3310–3318. 26685852 10.1007/s00330-015-4155-8

[2] Wong GTC, Lee EYP, Irwin MG. Contrast induced nephropathy in vascular surgery. Br J Anaesth. 2016;117:ii63–ii73. 27566809 10.1093/bja/aew213

[3] Betoko A, Matheson MB, Ostovaneh MR, et al. Acute kidney injury after repeated exposure to contrast material for coronary angiography. Mayo Clin Proc Innov Qual Outcomes. 2021;5(1):46–54. 33718783 10.1016/j.mayocpiqo.2020.08.012 PMC7930798

[4] Fähling M, Seeliger E, Patzak A, et al. Understanding and preventing contrast-induced acute kidney injury. Nat Rev Nephrol. 2017;13(3):169–180. 28138128 10.1038/nrneph.2016.196

[5] Stevens PE, Levin A. Evaluation and management of chronic kidney disease: synopsis of the kidney disease: improving global outcomes 2012 clinical practice guideline. Ann Intern Med. 2013;158(11):825–830. 23732715 10.7326/0003-4819-158-11-201306040-00007

[6] Lameire N, Kellum JA. Contrast-induced acute kidney injury and renal support for acute kidney injury: a KDIGO summary (part 2). Crit Care. 2013;17(1):205. 23394215 10.1186/cc11455 PMC4056805

[7] Nash K, Hafeez A, Hou S. Hospital-acquired renal insufficiency. Am J Kidney Dis. 2002;39(5):930–936. 11979336 10.1053/ajkd.2002.32766

[8] Morcos R, Kucharik M, Bansal P, et al. Contrast-induced acute kidney injury: review and practical update. Clin Med Insights Cardiol. 2019;13:1179546819878680. 31700251 10.1177/1179546819878680 PMC6826945

[9] Lakhal K, Robert-Edan V, Ehrmann S. In the name of contrast-induced acute kidney injury. Chest. 2020;157(4):751–752. 32252919 10.1016/j.chest.2019.12.009

[10] Xu Z, Zhang Y, Zhang C, et al. Clinical features and outcomes of COVID-19 patients with acute kidney injury and acute kidney injury on chronic kidney disease. Aging Dis. 2022;13(3):884–898. 35656097 10.14336/AD.2021.1125 PMC9116918

[11] Abdalla MA, Ahmed KO, Yousef BA. Incidence and risk factors of contrast-induced acute kidney injury in Sudanese patients undergoing coronary angiography: a descriptive prospective study. Cureus. 2022;14(2):e21876. 35273847 10.7759/cureus.21876 PMC8901158

[12] Husain-Syed F, Rosner MH, Ronco C. Distant organ dysfunction in acute kidney injury. Acta Physiol. 2020;228(2):e13357. 10.1111/apha.13357 31379123

[13] Kusirisin P, Chattipakorn SC, Chattipakorn N. Contrast-induced nephropathy and oxidative stress: mechanistic insights for better interventional approaches. J Transl Med. 2020;18(1):400. 33081797 10.1186/s12967-020-02574-8 PMC7576747

[14] Bansal S, Patel RN. Pathophysiology of contrast-induced acute kidney injury. Interv Cardiol Clin. 2020;9(3):293–298. 32471670 10.1016/j.iccl.2020.03.001

[15] Faucon AL, Bobrie G, Clement O. Nephrotoxicity of iodinated contrast media: from pathophysiology to prevention strategies. Eur J Radiol. 2019;116:231–241. 31054788 10.1016/j.ejrad.2019.03.008

[16] Ma C, Chen T, Ti Y, et al. Ranolazine alleviates contrast-associated acute kidney injury through modulation of calcium independent oxidative stress and apoptosis. Life Sci. 2021;267:118920. 33352171 10.1016/j.lfs.2020.118920

[17] Yang KJ, Kim JH, Chang YK, et al. Inhibition of xanthine oxidoreductase protects against contrast-induced renal tubular injury by activating adenosine monophosphate-activated protein kinase. Free Radic Biol Med. 2019;145:209–220. 31560952 10.1016/j.freeradbiomed.2019.09.027

[18] Williams AR, Wiggins RC, Wharram BL, et al. Nephron injury induced by diagnostic ultrasound imaging at high mechanical index with gas body contrast agent. Ultrasound Med Biol. 2007;33(8):1336–1344. 17507144 10.1016/j.ultrasmedbio.2007.03.002 PMC1986772

[19] Moore JK, Chen J, Pan H, et al. Quantification of vascular damage in acute kidney injury with fluorine magnetic resonance imaging and spectroscopy. Magn Reson Med. 2018;79(6):3144–3153. 29148253 10.1002/mrm.26985 PMC5897913

[20] Ghumman SS, Weinerman J, Khan A, et al. Contrast induced-acute kidney injury following peripheral angiography with carbon dioxide versus iodinated contrast media: a meta-analysis and systematic review of current literature. Catheter Cardiovasc Interv. 2017;90(3):437–448. 28463460 10.1002/ccd.27051

[21] van der Molen AJ, Reimer P, Dekkers IA, et al. Post-contrast acute kidney injury. Part 2: risk stratification, role of hydration and other prophylactic measures, patients taking metformin and chronic dialysis patients: recommendations for updated ESUR contrast medium safety committee guidelines. Eur Radiol. 2018;28(7):2856–2869. 29417249 10.1007/s00330-017-5247-4 PMC5986837

[22] Ward DB, Valentovic MA. Contrast induced acute kidney injury and direct cytotoxicity of iodinated radiocontrast media on renal proximal tubule cells. J Pharmacol Exp Ther. 2019;370(2):160–171.3110168010.1124/jpet.119.257337

[23] Sugimoto CR, Ahn Y-Y, Smith E, et al. Factors affecting sex-related reporting in medical research: a cross-disciplinary bibliometric analysis. Lancet. 2019;393(10171):550–559. 30739690 10.1016/S0140-6736(18)32995-7

[24] Martin-Martin A, Thelwall M, Orduna-Malea E, et al. Google Scholar, Microsoft Academic, Scopus, Dimensions, Web of Science, and OpenCitations’ COCI: a multidisciplinary comparison of coverage via citations. Scientometrics. 2021;126(1):871–906. 32981987 10.1007/s11192-020-03690-4 PMC7505221

[25] Chen C, CiteSpace II. Detecting and visualizing emerging trends and transient patterns in scientific literature. J Am Soc Inform Sci Technol. 2006;57(3):359–377.

[26] van Eck NJ, Waltman L. Citation-based clustering of publications using CitNetExplorer and VOSviewer. Scientometrics. 2017;111(2):1053–1070. 28490825 10.1007/s11192-017-2300-7 PMC5400793

[27] Tan L, Wang X, Yuan K, et al. Structural and temporal dynamics analysis on drug-eluting stents: history, research hotspots and emerging trends. Bioact Mater. 2023;23:170–186. 36406256 10.1016/j.bioactmat.2022.09.009 PMC9663333

[28] Wang H, Wang Q, Hu J, et al. Global research trends in in-stent neoatherosclerosis: a CiteSpace-based visual analysis. Front Cardiovasc Med. 2022;9:1025858. 36426225 10.3389/fcvm.2022.1025858 PMC9679497

[29] Khwaja A. KDIGO clinical practice guidelines for acute kidney injury. Nephron Clin Pract. 2012;120(4):c179–c184. 22890468 10.1159/000339789

[30] Levine GN, Bates ER, Blankenship JC, et al. 2011 ACCF/AHA/SCAI guideline for percutaneous coronary intervention: executive summary: a report of the American College of Cardiology Foundation/American Heart Association Task Force on practice guidelines and the Society for Cardiovascular Angiography and Interventions. Circulation. 2011;124(23):2574–2609. 22064598 10.1161/CIR.0b013e31823a5596

[31] Mehran R, Aymong ED, Nikolsky E, et al. A simple risk score for prediction of contrast-induced nephropathy after percutaneous coronary intervention: development and initial validation. J Am Coll Cardiol. 2004;44(7):1393–1399. 15464318 10.1016/j.jacc.2004.06.068

[32] Aboyans V, Ricco JB, Bartelink ML, et al. [2017 ESC guidelines on the diagnosis and treatment of peripheral arterial diseases, in collaboration with the European Society for Vascular Surgery (ESVS)]. Kardiol Pol. 2017;75(11):1065–1160. 29589371 10.5603/KP.2017.0216

[33] Kellum JA, Lameire N. Diagnosis, evaluation, and management of acute kidney injury: a KDIGO summary (part 1). Crit Care. 2013;17(1):204. 23394211 10.1186/cc11454 PMC4057151

[34] Kushner FG, Hand M, Smith SC Jr., et al. 2009 Focused updates: ACC/AHA guidelines for the management of patients with ST-Elevation myocardial infarction (updating the 2004 guideline and 2007 focused update) and ACC/AHA/SCAI guidelines on percutaneous coronary intervention (updating the 2005 guideline and 2007 focused update): a report of the American College of Cardiology Foundation/American Heart Association Task Force on practice guidelines. Circulation. 2009;120(22):2271–2306. 19923169 10.1161/CIRCULATIONAHA.109.192663

[35] Kushner FG, Hand M, Smith SC Jr., et al. 2009 Focused updates: ACC/AHA guidelines for the management of patients with ST-elevation myocardial infarction (updating the 2004 guideline and 2007 focused update) and ACC/AHA/SCAI guidelines on percutaneous coronary intervention (updating the 2005 guideline and 2007 focused update) a report of the American College of Cardiology Foundation/American Heart Association Task Force on practice guidelines. J Am Coll Cardiol. 2009;54(23):2205–2241. 19942100 10.1016/j.jacc.2009.10.015

[36] KDOQI. Clinical practice guidelines and clinical practice recommendations for diabetes and chronic kidney disease. Am J Kidney Dis. 2007;49(2 Suppl 2):S12–S154. 17276798 10.1053/j.ajkd.2006.12.005

[37] Bellomo R, Kellum JA, Ronco C. Acute kidney injury. Lancet. 2012;380(9843):756–766. 22617274 10.1016/S0140-6736(11)61454-2

[38] Merten GJ, Burgess WP, Gray LV, et al. Prevention of contrast-induced nephropathy with sodium bicarbonate: a randomized controlled trial. JAMA. 2004;291(19):2328–2334. 15150204 10.1001/jama.291.19.2328

[39] Nijssen EC, Rennenberg RJ, Nelemans PJ, et al. Prophylactic hydration to protect renal function from intravascular iodinated contrast material in patients at high risk of contrast-induced nephropathy (AMACING): a prospective, randomised, phase 3, controlled, open-label, non-inferiority trial. Lancet. 2017;389(10076):1312–1322. 28233565 10.1016/S0140-6736(17)30057-0

[40] Briguori C, Airoldi F, D'Andrea D, et al. Renal insufficiency following contrast media administration trial (REMEDIAL): a randomized comparison of 3 preventive strategies. Circulation. 2007;115(10):1211–1217. 17309916 10.1161/CIRCULATIONAHA.106.687152

[41] McCullough PA. Contrast-induced acute kidney injury. J Am Coll Cardiol. 2008;51(15):1419–1428. 18402894 10.1016/j.jacc.2007.12.035

[42] Marenzi G, Assanelli E, Marana I, et al. *N*-acetylcysteine and contrast-induced nephropathy in primary angioplasty. N Engl J Med. 2006;354(26):2773–2782. 16807414 10.1056/NEJMoa054209

[43] Weisbord SD, Gallagher M, Jneid H, et al. Outcomes after angiography with sodium bicarbonate and acetylcysteine. N Engl J Med. 2018;378(7):603–614. 29130810 10.1056/NEJMoa1710933

[44] Mehran R, Dangas GD, Weisbord SD. Contrast-associated acute kidney injury. N Engl J Med. 2019;380(22):2146–2155. 31141635 10.1056/NEJMra1805256

[45] Aspelin P, Aubry P, Fransson SG, et al. Nephrotoxic effects in high-risk patients undergoing angiography. N Engl J Med. 2003;348(6):491–499. 12571256 10.1056/NEJMoa021833

[46] Stacul F, van der Molen AJ, Reimer P, et al. Contrast induced nephropathy: updated ESUR contrast media safety committee guidelines. Eur Radiol. 2011;21(12):2527–2541. 21866433 10.1007/s00330-011-2225-0

[47] Kelly AM, Dwamena B, Cronin P, et al. Meta-analysis: effectiveness of drugs for preventing contrast-induced nephropathy. Ann Intern Med. 2008;148(4):284–294. 18283206 10.7326/0003-4819-148-4-200802190-00007

[48] Subramaniam RM, Suarez-Cuervo C, Wilson RF, et al. Effectiveness of prevention strategies for Contrast-Induced nephropathy: a systematic review and meta-analysis. Ann Intern Med. 2016;164(6):406–416. 26830221 10.7326/M15-1456

[49] Benko A, Fraser-Hill M, Magner P, et al. Canadian association of radiologists: consensus guidelines for the prevention of contrast-induced nephropathy. Can Assoc Radiol J. 2007;58(2):79–87. 17521052

[50] Meschi M, Detrenis S, Savazzi G. [Contrast-induced nephropathy. Current concepts and propositions for Italian guidelines]. Recenti Prog Med. 2008;99(3):155–162. 18488528

[51] Joannidis M, Druml W, Forni LG, et al. Prevention of acute kidney injury and protection of renal function in the intensive care unit. Expert opinion of the working group for nephrology, ESICM. Intensive Care Med. 2010;36(3):392–411. 19921152 10.1007/s00134-009-1678-y

[52] Reed MC, Moscucci M, Smith DE, et al. The relative renal safety of iodixanol and low-osmolar contrast media in patients undergoing percutaneous coronary intervention. Insights from blue cross blue shield of Michigan Cardiovascular Consortium (BMC2). J Invasive Cardiol. 2010;22(10):467–472. 20944185

[53] Ronco C, Maioli M, Lorusso V, et al. [Contrast-induced nephropathy: the VIKISAFE study group statement]. G Ital Nefrol. 2012;29(2):183–204. 22538948

[54] Ad-Hoc Working Group of EEBP, Fliser D, Laville M, et al. A European Renal Best Practice (ERBP) position statement on the kidney disease improving global outcomes (KDIGO) clinical practice guidelines on acute kidney injury: part 1: definitions, conservative management and contrast-induced nephropathy. Nephrol Dial Transplant. 2012;27(12):4263–4272. 23045432 10.1093/ndt/gfs375 PMC3520085

[55] Ohno I, Hayashi H, Aonuma K, et al. Guidelines on the use of iodinated contrast media in patients with kidney disease 2012: digest version. JSN, JRS, and JCS joint working group. Jpn J Radiol. 2013;31(8):546–584. 23884513 10.1007/s11604-013-0226-4

[56] Owen RJ, Hiremath S, Myers A, et al. Canadian association of radiologists consensus guidelines for the prevention of contrast-induced nephropathy: update 2012. Can Assoc Radiol J. 2014;65(2):96–105. 24559602 10.1016/j.carj.2012.11.002

[57] Levine GN, Bates ER, Blankenship JC, et al. 2011 ACCF/AHA/SCAI guideline for percutaneous coronary intervention: a report of the American College of Cardiology Foundation/American Heart Association Task Force on practice guidelines and the Society for Cardiovascular Angiography and Interventions. Circulation. 2011;124(23):e574–e651. 22064601 10.1161/CIR.0b013e31823ba622

[58] Berkseth RO, Kjellstrand CM. Radiologic contrast-induced nephropathy. Med Clin North Am. 1984;68(2):351–370. 6423916 10.1016/s0025-7125(16)31135-x

[59] van der Molen AJ, Reimer P, Dekkers IA, et al. Post-contrast acute kidney injury – part 1: definition, clinical features, incidence, role of contrast medium and risk factors: recommendations for updated ESUR Contrast Medium Safety Committee guidelines. Eur Radiol. 2018;28(7):2845–2855. 29426991 10.1007/s00330-017-5246-5 PMC5986826

[60] de Laforcade L, Bobot M, Bellin MF, et al. Kidney and contrast media: common viewpoint of the French Nephrology Societies (SFNDT, FIRN, CJN) and the French Radiological Society (SFR) following ESUR guidelines. Diagn Interv Imaging. 2021;102(3):131–139. 33531265 10.1016/j.diii.2021.01.007

[61] Davenport MS, Perazella MA, Yee J, et al. Use of intravenous iodinated contrast media in patients with kidney disease: consensus statements from the American College of Radiology and the National Kidney Foundation. Radiology. 2020;294(3):660–668. 31961246 10.1148/radiol.2019192094

[62] Davenport MS, Cohan RH, Khalatbari S, et al. The challenges in assessing contrast-induced nephropathy: where are we now? Am J Roentgenol. 2014;202(4):784–789. 24660707 10.2214/AJR.13.11369

[63] Kodzwa R. ACR manual on contrast media: 2018 updates. Radiol Technol. 2019;91(1):97–100. 31471485

[64] Lautin EM, Freeman NJ, Schoenfeld AH, et al. Radiocontrast-associated renal dysfunction: a comparison of lower-osmolality and conventional high-osmolality contrast media. Am J Roentgenol. 1991;157(1):59–65. 2048540 10.2214/ajr.157.1.2048540

[65] Ren L, Wang P, Wang Z, et al. Hypotonic contrast media is more toxic than isotonic contrast media on endothelial cells *in vivo* and *in vitro*. Mol Med Rep. 2017;16(4):4334–4340. 28731176 10.3892/mmr.2017.7066

[66] Dangas G, Iakovou I, Nikolsky E, et al. Contrast-induced nephropathy after percutaneous coronary interventions in relation to chronic kidney disease and hemodynamic variables. Am J Cardiol. 2005;95(1):13–19. 15619387 10.1016/j.amjcard.2004.08.056

[67] Rihal CS, Textor SC, Grill DE, et al. Incidence and prognostic importance of acute renal failure after percutaneous coronary intervention. Circulation. 2002;105(19):2259–2264. 12010907 10.1161/01.cir.0000016043.87291.33

[68] Tong GE, Kumar S, Chong KC, et al. Risk of contrast-induced nephropathy for patients receiving intravenous vs. intra-arterial iodixanol administration. Abdom Radiol. 2016;41(1):91–99. 10.1007/s00261-015-0611-9 26830615

[69] McDonald JS, Leake CB, McDonald RJ, et al. Acute kidney injury after intravenous versus intra-arterial contrast material administration in a paired cohort. Invest Radiol. 2016;51(12):804–809. 27299579 10.1097/RLI.0000000000000298

[70] Chaudhury P, Armanyous S, Harb SC, et al. Intra-arterial versus intravenous contrast and renal injury in chronic kidney disease: a propensity-matched analysis. Nephron. 2019;141(1):31–40. 30368506 10.1159/000494047

[71] Schönenberger E, Martus P, Bosserdt M, et al. Kidney injury after intravenous versus intra-arterial contrast agent in patients suspected of having coronary artery disease: a randomized trial. Radiology. 2019;292(3):664–672. 31264950 10.1148/radiol.2019182220

[72] Gutierrez NM, Newhouse JH. Maximum arterial contrast concentrations with computed tomography and left ventriculography: implications for contrast nephrotoxicity risk. J Comput Assist Tomogr. 2017;41(6):976–982. 28481807 10.1097/RCT.0000000000000624

[73] Sgura FA, Bertelli L, Monopoli D, et al. Mehran contrast-induced nephropathy risk score predicts short- and long-term clinical outcomes in patients with ST-elevation-myocardial infarction. Circ Cardiovasc Interv. 2010;3(5):491–498. 20923986 10.1161/CIRCINTERVENTIONS.110.955310

[74] Wang Z, Ren K. Evaluation of iodine contrast-induced acute kidney injury via different injection routes using BOLD-MRI. Ren Fail. 2019;41(1):341–353. 31057054 10.1080/0886022X.2019.1604382 PMC6508059

[75] Zhang J, Guo Y, Jin Q, et al. Meta-analysis of rosuvastatin efficacy in prevention of contrast-induced acute kidney injury. Drug Des Devel Ther. 2018;12:3685–3690. 10.2147/DDDT.S178020 PMC6216974 30464400

[76] Wang XL, Zhang T, Hu LH, et al. Comparison of effects of different statins on contrast-induced acute kidney injury in rats: histopathological and biochemical findings. Oxid Med Cell Longev. 2017;2017:6282486. 28243357 10.1155/2017/6282486 PMC5294380

[77] Zhou X, Dai J, Xu X, et al. Comparative efficacy of statins for prevention of contrast-induced acute kidney injury in patients with chronic kidney disease: a network meta-analysis. Angiology. 2019;70(4):305–316. 30261736 10.1177/0003319718801246

[78] Hall KA, Wong RW, Hunter GC, et al. Contrast-induced nephrotoxicity: the effects of vasodilator therapy. J Surg Res. 1992;53(4):317–320. 1405611 10.1016/0022-4804(92)90054-4

[79] Jeong BY, Lee HY, Park CG, et al. Oxidative stress caused by activation of NADPH oxidase 4 promotes contrast-induced acute kidney injury. PLOS One. 2018;13(1):e0191034. 29329317 10.1371/journal.pone.0191034 PMC5766150

[80] Hussien NI, Sorour SM, El-Kerdasy HI, et al. The glucagon-like peptide-1 receptor agonist exendin-4, ameliorates contrast-induced nephropathy through suppression of oxidative stress, vascular dysfunction and apoptosis independent of glycaemia. Clin Exp Pharmacol Physiol. 2018;45(8):808–818. 29637584 10.1111/1440-1681.12944

[81] Lin HH, Lee TS, Lin SJ, et al. DDAH-2 alleviates contrast medium iopromide-induced acute kidney injury through nitric oxide synthase. Clin Sci. 2019;133(23):2361–2378. 10.1042/CS20190455 31763675

[82] Lin Q, Li S, Jiang N, et al. PINK1-parkin pathway of mitophagy protects against contrast-induced acute kidney injury via decreasing mitochondrial ROS and NLRP3 inflammasome activation. Redox Biol. 2019;26:101254. 31229841 10.1016/j.redox.2019.101254 PMC6597739

[83] Caiazza A, Russo L, Sabbatini M, et al. Hemodynamic and tubular changes induced by contrast media. Biomed Res Int. 2014;2014:578974. 24678510 10.1155/2014/578974 PMC3941595

[84] Heyman SN, Rosen S, Rosenberger C. Renal parenchymal hypoxia, hypoxia adaptation, and the pathogenesis of radiocontrast nephropathy. Clin J Am Soc Nephrol. 2008;3(1):288–296. 18057308 10.2215/CJN.02600607

[85] Lamby P, Krüger-Genge A, Franke RP, et al. Effect of iodinated contrast media on the oxygen tension in the renal cortico-medullary region of pigs. Clin Hemorheol Microcirc. 2019;73(1):261–270. 31322554 10.3233/CH-199009

