# Supplementary figures and images for: The intellectual base and global trends in contrast-induced acute kidney injury: a bibliometric analysis
Source: Ren Fail. 2023 Mar 17;45(1):2188967. doi: 10.1080/0886022X.2023.2188967 (PMC10026803; doi:10.1080/0886022X.2023.2188967)

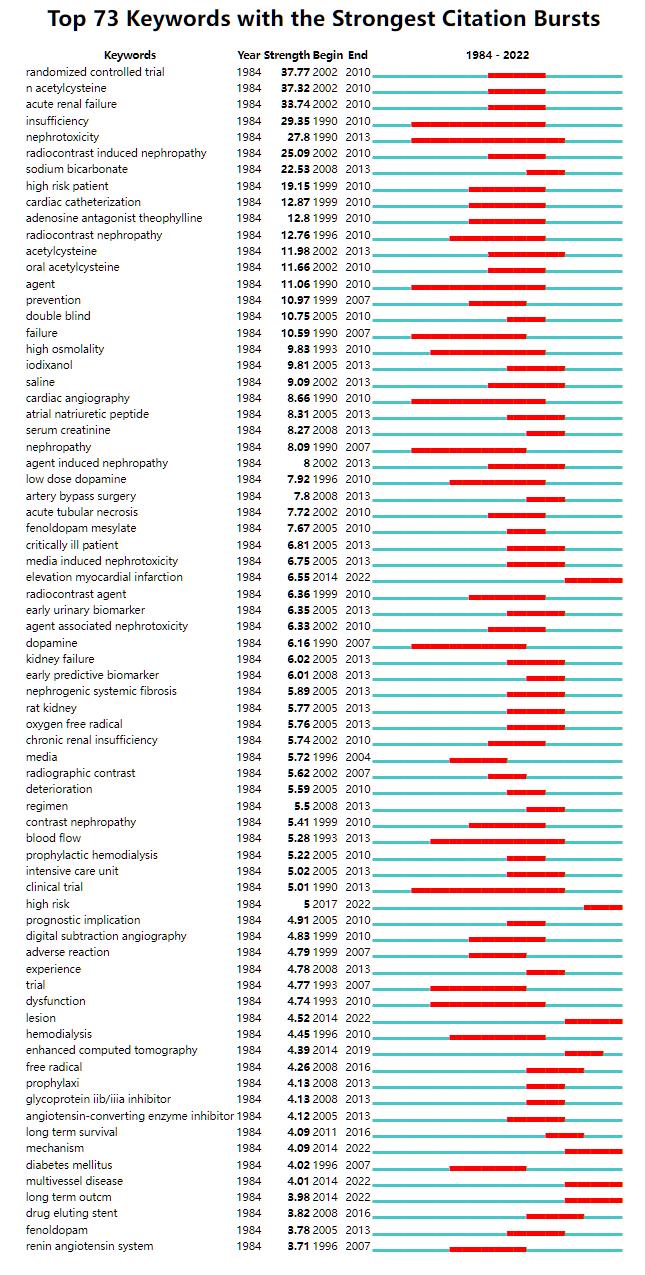

Supplement: Supplemental Material [file IRNF_A_2188967_SM4533.jpg]

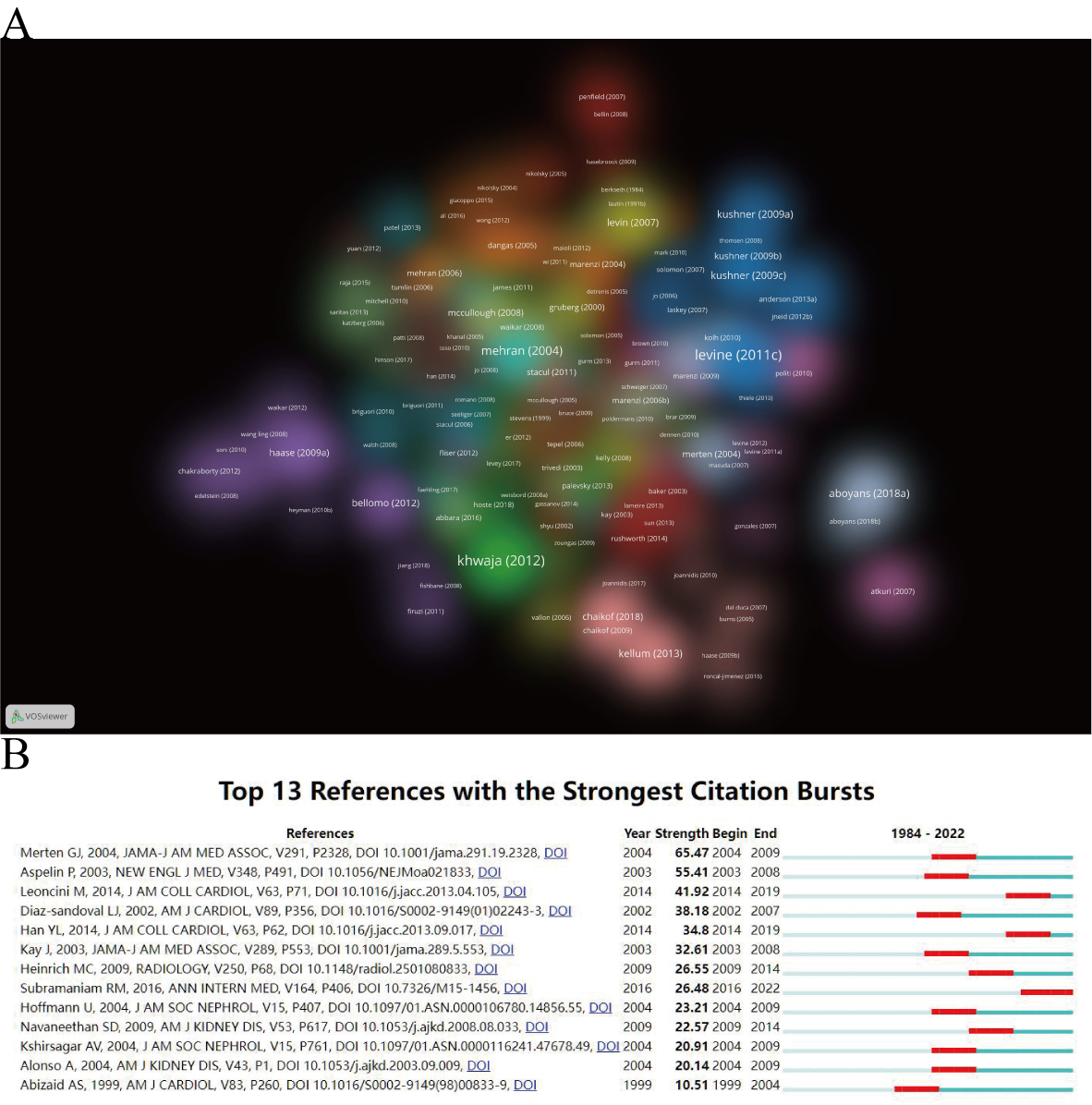

Supplement: Supplemental Material [file IRNF_A_2188967_SM4530.tif]

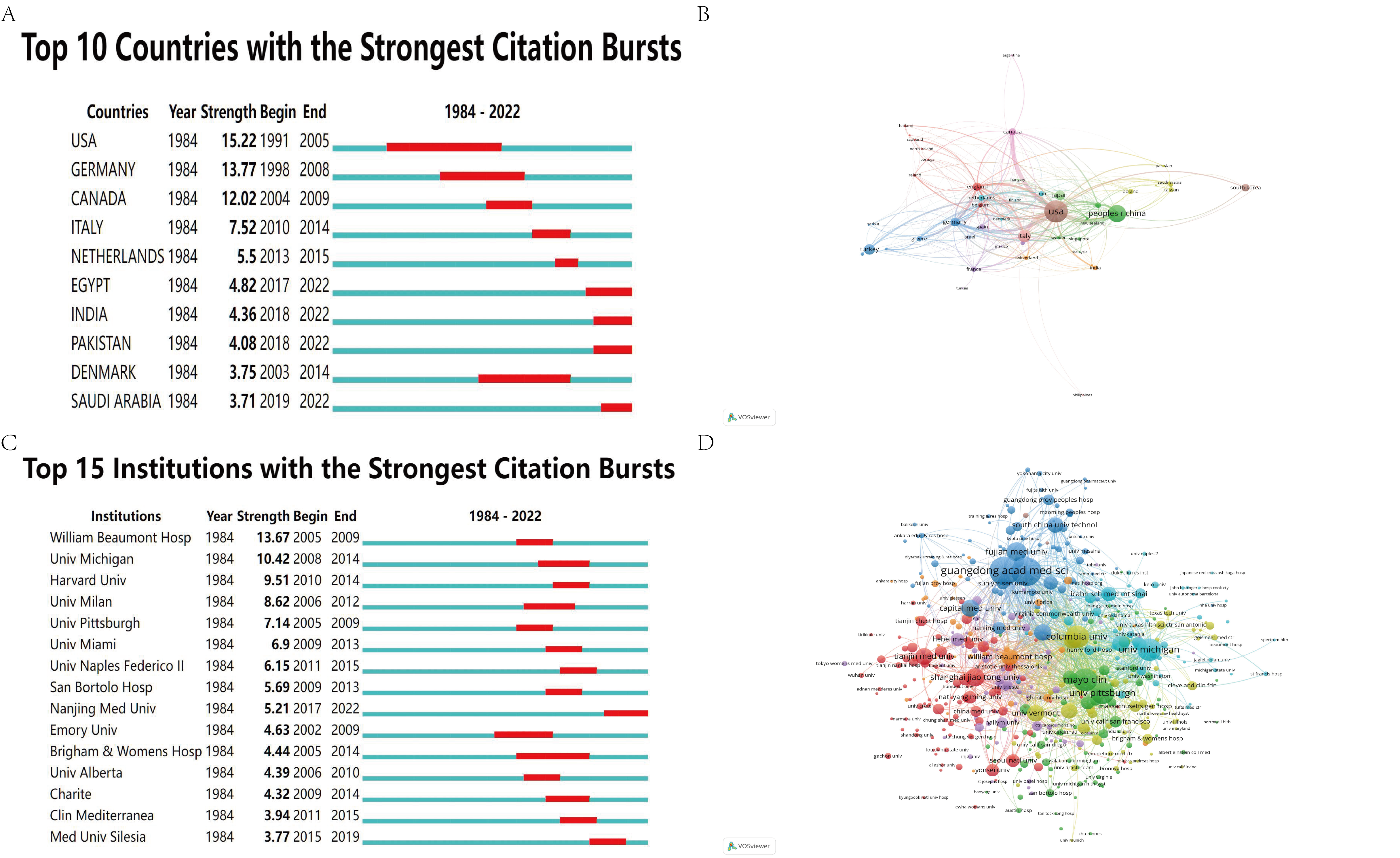

Supplement: Supplemental Material [file IRNF_A_2188967_SM4077.tif]
